# Hotspot mutations delineating diverse mutational signatures and biological utilities across cancer types

**DOI:** 10.1186/s12864-016-2727-x

**Published:** 2016-06-23

**Authors:** Tenghui Chen, Zixing Wang, Wanding Zhou, Zechen Chong, Funda Meric-Bernstam, Gordon B. Mills, Ken Chen

**Affiliations:** Department of Bioinformatics and Computational Biology, The University of Texas MD Anderson Cancer Center, Houston, TX 77030 USA; Department of Investigational Cancer Therapy, The University of Texas MD Anderson Cancer Center, Houston, TX 77030 USA; Department of Systems Biology, The University of Texas MD Anderson Cancer Center, Houston, TX 77030 USA; Institute for Personalized Cancer Therapy, The University of Texas MD Anderson Cancer Center, Houston, TX 77030 USA; Biostatistics, Bioinformatics and Systems Biology Program, The University of Texas Graduate School of Biomedical Sciences, Houston, TX 77030 USA

## Abstract

**Background:**

An important step towards personalizing cancer treatment is to integrate heterogeneous evidences to catalog mutational hotspots that are biologically and therapeutically relevant and thus represent where targeted therapy would likely be beneficial. However, existing methods do not sufficiently delineate varying functionality of individual mutations within the same genes.

**Results:**

We observed a large discordancy of mutation rates across different mutation subtypes and tumor types, and nominated 702 hotspot mutations in 549 genes in the Catalog of Somatic Mutations in Cancer (COSMIC) by considering context specific mutation characteristics such as genes, cancer types, mutation rates, mutation subtypes and sequence contexts. We observed that hotspot mutations were highly prevalent in Non CpG-island C/G transition and transversion sequence contexts in 10 tumor types, and specific insertion hotspot mutations were enriched in breast cancer and deletion hotspot mutations in colorectal cancer. We found that the hotspot mutations nominated by our approach were significantly more conserved than non-hotspot mutations in the corresponding cancer genes. We also examined the biological significance and pharmacogenomics properties of these hotspot mutations using data in the Cancer Genome Atlas (TCGA) and the Cancer Cell-Line Encyclopedia (CCLE), and found that 53 hotspot mutations are independently associated with diverse functional evidences in 1) mRNA and protein expression, 2) pathway activity, or 3) drug sensitivity and 82 were highly enriched in specific tumor types. We highlighted the distinct functional indications of hotspot mutations under different contexts and nominated novel hotspot mutations such as *MAP3K4* A1199 deletion, *NR1H2* Q175 insertion, and *GATA3* P409 insertion as potential biomarkers or drug targets.

**Conclusion:**

We identified a set of hotspot mutations across 17 tumor types by considering the background mutation rate variations among genes, tumor subtypes, mutation subtypes, and sequence contexts. We illustrated the common and distinct mutational signatures of hotspot mutations among different tumor types and investigated their variable functional relevance under different contexts, which could potentially serve as a resource for explicitly selecting targets for diagnosis, drug development, and patient management.

**Electronic supplementary material:**

The online version of this article (doi:10.1186/s12864-016-2727-x) contains supplementary material, which is available to authorized users.

## Background

One of the critical challenges of oncogenomics and pharmacogenomics is to distinguish genomic alterations that confer tumorigenesis (i.e. drivers), from those that provide no selective advantage to tumor growth but occur stochastically in cancer development. Although it becomes clear that genomic profiles obtained from clinical sequencing data can inform clinical decision making, the implementation of cancer genomic medicine is critically constrained by a lack of understanding of the impact of individual somatic mutations on tumor pathophysiology and response to cancer therapy under different disease contexts.

There were several methods that focused on predicting driver genes. A gene is nominated as a driver if it contains significantly more mutations than expected from a null background model [1, 2]. A variety of practical algorithms have been developed in the context of large-scale cancer genome sequencing, differing mainly by how they model background mutations. For example, MuSiC [3] considers the difference in mutation types but assumes a homogenous background mutation rate across all genes. MutSigCV [4] modeled heterogeneous background mutation rate as a function of gene, replication timing, sequence context, cancer type and epigenetic elements. OncodriveCLUST [5] estimates background model from coding-silent mutations and tests protein domains containing clusters of missense mutations that are likely to alter protein structure. E-Driver [6] uses protein 3D structural features to predict driver genes containing clusters of missense mutations in protein-protein interaction (PPI) interfaces. However, increasingly more studies indicate that a mutation may have substantially different functions at different amino acid positions in the same gene [7, 8] and may be associated with different clinical utilities in different disease and biological contexts [9, 10]. Additionally, those studies mostly ignored the potentially functional mutations in infrequently mutated genes, and in under-investigated mutation types such as insertions and deletions.

To date, the studies on hotspot mutations have been limited in individual cancer types [11, 12] or have assumed identical functions of mutations in the same genes [5, 6]. The number of clinically actionable mutations has been very limited (currently 285 in MyCancerGenome.org and 269 in PersonalizedCancerTherapy.org), and it is critical to systematically analyze hotspot mutations by performing genome-wide and population-based analysis across different tumor types and assessing functionality using RNA expression, protein activity and drug response data. As clinical sequencing becomes a central platform for achieving personalized therapy, obtaining accurate biological and therapeutic interpretation of a large number of mutations in a tumor type specific manner will greatly enhance the efficacy of genomics in clinical applications.

Toward the mutational signatures under different sequence contexts, previous studies [13, 14] have indicated sequence context mutation rate diversities across different cancer types and reported that C/G transitions such as C > T and C/G transversions such as C > A occupy a high proportion at single nucleotide variant level. Those investigations were mostly motivated from the perspective of understanding the mutational signatures that use all the observed mutations. It is interesting to investigate when focusing on potentially functional mutations such as hotspot mutations, whether the mutational signatures would be different after genomic positive selection and be enriched under different sequence contexts as compared to what was observed using all mutations. In addition, previous studies mostly focused on investigating single nucleotide variants but frequently ignore the small insertions and deletions [13], which represent a significant part of functional mutations.

In this study, we defined a hotspot mutation as a mutation that occurs in a set of tumor samples significantly more frequently than expected from a background frequency characterized by genes, cancer types, mutation types and sequence contexts. We investigated the mutational signatures of hotspot mutations and illustrated the common and distinct sequence contexts under which the hotspot mutations were enriched across different tumor types. We also investigated and revealed substantial functional variations of hotspot mutations under different contexts and nominated a set of novel hotspot mutations, which could potentially serve as a resource for precisely selecting targets for diagnosis, drug development, and patient management.

## Methods

### COSMIC somatic mutation data

We downloaded the COSMIC somatic mutation dataset version 71 for our study. This set (12,250 samples) includes many sources of curated mutation data. We excluded samples that underwent targeted-sequencing [15], and selected only those that were subjected to either whole genome or whole exome sequencing (Additional file 1: Table S1). In this manner, we ensured that all the exons of investigated genes were uniformly examined in the selected samples.

### Cancer gene candidates

We collected from literature a set of candidate cancer genes, which included 546 genes reported in cancer gene census [16], 435 genes in Pancan12 [17], 221 genes reported in Lawrence et al. [18]. For OncodriveCLUST [5] and e-Driver [6], we applied their algorithms to predict tumor type-specific driver genes using COSMIC v71 mutation data. We used q-value < 0.01 and q-value < 0.05 to determine driver genes in OncodriveCLUST and in e-Driver, respectively.

### Definition of hotspot mutations

**Fig. 1:**
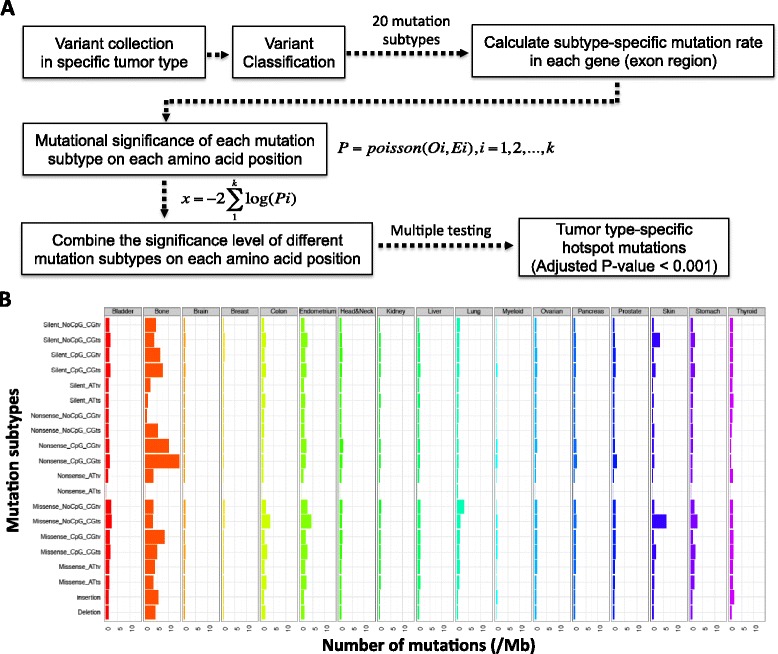
Statistics of the mutation distribution in different tumor types in COSMIC and an overview of HotDriver. **a** Providing a mutational profile from a specific tumor type, the variants were classified into 20 mutation subtypes, then the mutation subtype-specific mutations rates were computed for each investigated genes and the significant level of each amino acid position on the corresponding genes was calculated. After that, the significant level of each amino acid position was calculated by combining *p* values from different mutation subtypes using Fisher’s method, and an adjusted *p* value was computed for each amino acid position. **b** The mutation rate of 20 mutation subtypes in 17 main tumor types of COSMIC v71 whole genome and whole exome sequencing data

Our algorithm identifies hotspots based on amino acid (AA) positions (Fig. 1a). Five major mutation types were included in our modeling: missense, nonsense, coding-silent, insertion and deletion. For missense, nonsense and coding-silent mutations, six types of sequence context were considered: A/T transition (ATts), A/T transversion (ATtv), CpG G/C transition (CpG_CGts), non-CpG G/C transition (NoCpG_CGts), CpG G/C transversion (CpG_CGtv), non-CpG G/C transversion (NoCpG_CGtv), as previously introduced [3]. Altogether, 20 mutation subtypes were considered (Additional file 2: Table S2). For each mutation subtype in each gene, we counted the number of subtype-specific mutations across all the samples. For each gene, we calculated the mean subtype-specific mutation rate as the total number of subtype-specific mutations in the coding regions *(E)* divided (normalized) by the protein length. We calculated a *p*-value based on the number of observed subtype-specific mutations *(O)* in a given AA, assuming the number of mutations in each mutation subtype follows a Poisson distribution. After obtaining a *p*-value for each mutation subtype, we computed an integrated *p*-value for each AA based on Fisher’s method [19]

$$ x=-2{\displaystyle {\sum}_{i=1}^k log\left( pois\left({O}_i,{E}_i\right)\right),} $$

where *i* represents a mutation subtype, and *pois* the Poisson distribution; *x* follows a chi-square distribution with 2 *k* degrees of freedom, where *k* is the number of mutation subtypes tested. We further applied false discovery rate correction [20] and reported hotspot mutations in AA positions with adjusted *p*-value < 0.001 in COSMIC.

### TCGA pan-cancer data

We downloaded TCGA pan-cancer level-3 somatic mutation, copy number alteration and RNA expression data from Synapse (https://www.synapse.org/#!Synapse:syn300013), and RPPA data from TCPA (http://app1.bioinformatics.mdanderson.org/tcpa/_design/basic/index.html) [21]. More than 4400 tumor samples were assayed by whole exome sequencing, total RNA sequencing [22], or reverse phase protein array (RPPA) technologies. The number of tumor samples available for each cancer type is listed in Additional file 3: Table S3. We called deletions where the normalized estimated copy value is less than −1 and amplifications where the value is greater than 1. We used the normalized TCGA level-3 RNA expression data in our study. To allow for log transformation, the RPKM values of 0 were set to the minimum nonzero RPKM in the given samples. We applied log_2_ transformation to all mRNA RPKM expression values, as described by Jacobsen et al. [23]. We analyzed 181 proteins in total using RPPA, which contains 181 high-quality antibodies targeting 128 total proteins and 53 post-translationally modified proteins. We used the normalized level-3 RPPA data (level-4 data for Breast invasive carcinoma) in our study [21].

To test association between mutations and RNA expressions, we used samples that had available both somatic mutations and RNA expression data. To test association between mutations and protein expressions, we used samples that had available both somatic mutation and RPPA data (see Additional file 3: Table S3).

### Cancer Cell Line Encyclopedia (CCLE) mutation and drug sensitivity data

The CCLE [24] contains drug activity data of 24 different compounds in 504 cell lines and somatic mutation data of 906 cell lines. In our analysis, we included cell lines with both drug sensitivity and mutation data. Drug sensitivity data were adjusted by a logistical-sigmoidal function and described by 4 different variables: the maximal effect level (Amax), the drug concentration at half-maximal activity of the compound (EC50), the concentration at which the drug response reached an absolute inhibition of 50 % (IC50), and the activity area, which is the area above the dose–response curve [24]. In our analysis, we used the activity area, which captures both efficacy and potency of drug activity according to the CCLE, to measure drug responses.

### Tumor-type prevalence of hotspot mutations

To measure the prevalence of a hotspot mutation in tumor type ***A***, we calculated the number of ***A*** samples that contain a target mutation ***B***, the number of ***A*** samples that do not contain ***B***, the number of non-***A*** samples that contain ***B***, and the number of non-***A*** samples that do not contain ***B***, respectively (Additional file 4: Table S4). Then we used Fisher’s exact test to compute the significance and applied FDR correction. A hotspot is called highly prevalent in a specific tumor type if its adjusted *p*-value < 0.01.

### Conservation score comparison

We downloaded the chromosomal base-wise Genomic Evolutionary Rate Profiling (GERP) scores computed by GERP++ [25]. In our study, we extracted the resistant substitution (RS) scores from the nucleotide bases that belong to hotspot mutations and that belong to non-hotspot mutations, and tested if the scores between these two groups were significantly different. A higher RS score represents stronger evolutionary conservation.

## Results

### Variable mutation rates among different tumor types and mutation subtypes

As mentioned previously (Methods), we classified all the mutations into 20 subtypes based on both mutation types and di-nucleotide sequence contexts (Additional file 2: Table S2). In the COSMIC mutation dataset, skin, stomach, bladder and colon tumors have relatively high overall mutational rates, which were consistent with a previous report [4]. Besides, we also observed high mutational rates in bone and endometrium tumors (Fig. 1b). However, we observed highly variable mutational rates across different mutation subtypes (Kruskal-Wallis H-test, *p* = 2.22e-05). For example, in bone tumors, nonsense non-CpG C/G transversion has a mutation rate of 0.69/Mb while nonsense CpG C/G transition has a mutation rate of 14.2/Mb. Similarly, the mutational rate can vary substantially across different tumor types (Kruskal-Wallis H-test, *p* = 3.49e-40). For example, missense non-CpG C/G transition has an average rate of 6.18/Mb in skin tumors, much higher than which in brain tumors (0.61/Mb). Therefore, to identify potentially drivers that are positively selected in cancer, it is important to account for variations in mutation subtype and sequence context in different tumor types, instead of examining only variant frequencies in the population.

### Identifying hotspot mutations in COSMIC

**Fig. 2:**
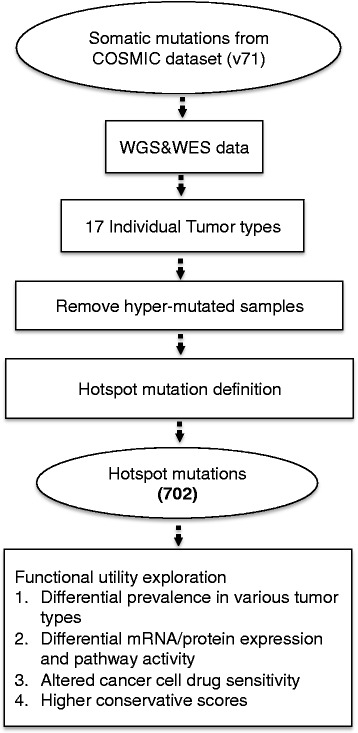
Illustration of hotspot mutations definition and functional utility analysis. We used COSMIC v71 data as the input. We first selected the samples that were examined with whole genome or whole exome sequencing, and then removed the hyper-mutated samples in each tumor types. Hotspot mutations were identified in individual tumor types, and the biological utility investigations were performed through multiple aspects

We started with all the mutations in 17 tumor types in COSMIC v71 (Fig. 2). Only data that were obtained via either whole exome or whole genome sequencing were used (Methods, Additional file 1: Table S1) [15]. Estimation of background mutation rates may be biased by outlier hyper-mutated samples. To avoid such bias, we calculated the mean μ and the standard deviation σ of the number of mutations in each sample, labeled the samples with numbers of mutations greater than μ + 2σ as hyper-mutated, and excluded them from further considerations (Additional file 1: Table S1).

Our goal was to identify hotspot mutations within genes (Methods) and to explore their potentially biological utilities under different biological contexts. The large number of samples in COSMIC made it possible to reliably estimate a background mutation rate for each gene in each tumor type and mutation subtype (Methods). We identified a hotspot mutation as the set of genomic aberrations that affect an amino acid (AA) position and occur significantly more frequently than expected from the background. In total, we identified a set of 702 putative hotspot mutations in 549 genes in 17 tumor types (Fig. 2, Methods).

We measured the composition of different mutational subtypes in the hotspot mutations (Additional file 5: Figure S1). As expected, 510 (72.65 %) were missense and 17 (2.42 %) were nonsense, occupying a high proportion of hotspot mutations. We also identified 31 insertion (4.42 %) and 78 deletion (11.11 %) hotspots, which were largely ignored in previously studies [5, 6] and potentially offered novel candidates for driver mutation and cancer gene prediction. Besides, we examined the insertion and deletion hotspots and found that 17/31 was in-frame insertions and 17/78 was in-frame deletions. Among the remaining frame-shift insertion and deletions hotspots, more than 70 % have slightly different start positions and/or sizes. For example, the ESRP1 N512 hotspot deletion has two genomic variants chr8:95686611A/- and chr8:95686611-95686612AA/-.

We found that the hotspot-mutation-containing-genes (HMCGs) identified in our study overlapped significantly (98/546 vs 451/24405, Fisher exact test, *p* = 1.28e-53) with the 546 cancer genes reported in the Caner Gene Census (CGC). Among 24,951 available genes in COSMIC, 549 genes were identified to contain at least one hotspot, among which 98 were the CGC cancer genes. Similarly, we found that HMCGs overlapped significantly with the significantly mutated genes reported in TCGA PANCAN analysis (101/435 vs 448/24516, Fisher exact test, *p* = 6.56e-74) and in Lawrence et al. (73/221 vs 476/24630, Fisher exact test, *p* = 2.56e-65). The non-overlapping genes were detected due likely to that 1) the previous studies had different background mutation rate assumptions than our study; 2) they detected large number of tumor suppressors that do not contain clear hotspot mutations; 3) our study was not only able to detect hotspot mutations in known cancer genes, but also capable of detecting hotspot mutations in infrequently mutated genes, which may have previously unknown biological functionality; 4) our study included mutation types (indels) that previous studies did not. The extent of overlap between HMCGs and the union of the above mentioned cancer gene sets remained highly significant when we chose various adjusted *p* value cutoffs to identify the hotspot mutations (Additional file 6: Figure S2), which indicated the statistical robustness of our approach.

Furthermore, we found significantly overlapped genes between our set with those predicted by other cluster-based methods such as e-Driver [6] (151/552 vs 398/24499, Fisher exact test, *p* = 3.42e-139) and OncodriveCLUST [5] (106/489 vs 443/24462, Fisher exact test, *p* = 2.31e-74). Additionally, regarding the mutational clusters, we found 213 hotspots overlapped with 1125 significant mutational clusters as identified by e-Driver (213/1125 vs 489/92822, Proportional test, *p* = 2.14e-87) and 261 hotspots overlapped with 1042 significant mutational clusters as predicted by OncodriveCLUST (261/1042 vs 441/89561, Proportional test, *p* = 4.98e-121). Non-overlapping results were found due mainly to: 1) e-Driver and OncodriveCLUST predicted clusters based mainly on missense mutations in a uniform mutational background; 2) our study identified not only missense hotspot mutations but also a substantial proportion of insertion (4.42 %) and deletion (11.11 %) hotspots (Additional file 5: Figure S1); 3) our study chose a more stringent statistical significance cutoff to increase the confidence of identified hotspot mutations.

**Fig. 3:**
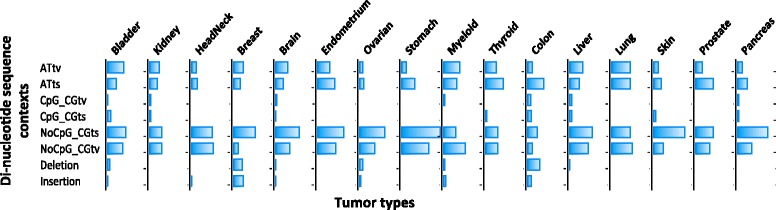
Mutational signatures of hotspot mutations in 16 tumor types. The x-axis represents the tumor types and the y-axis represent the 8 types of sequence contexts (concatenating missense, nonsense and silent mutations). Each bar represents the percentage of specific sequence contexts under which the hotspot mutations happen. In each tumor type, the addition of the percentages of different sequence contexts might be larger than 1, because one or more types of mutations may happen on a single hotspot driver mutation site

The number of hotspot mutations varied to a great extent from one tumor type to another (Additional file 7: Figure S3 and Additional file 8: Table S5). Most tumor types had 5 to 100 hotspot mutations. However, colorectal cancer had 253 hotspot mutations despite its relatively small sample size (684 samples), including a high proportion of insertion (10 %) and deletion (23 %) hotspot mutations (Fig. 3). In contrast, only 65 hotspot mutations were found in myeloid cancer (1344 samples). Such enrichment may reflect a higher extent of genetic heterogeneity in the initiation and progression of colorectal cancer, as has been suggested previously [26, 27] and also that colorectal cancer is predominantly driven by mutations rather than by copy number alterations [28]. In addition, we examined the numbers of hotspot mutations and the total numbers of mutations (mutation burden) in each tumor type, but did not find a clear correlation between them (Additional file 9: Figure S4).

### Sequence context signature of hotspot mutations

We investigated the mutational signatures of 702 hotspot mutations under different sequence contexts across different tumor types. As shown in Fig. 3, in 7 different tumor types (stomach, ovarian, brain, breast, skin, pancreas and kidney cancer), NoCpG_CGts was the most prevalent sequence context compared to other sequence contexts under which the hotspot mutations happened (*p* < 0.05), indicating a higher strength of positive selection on DNA sequences with NoCpG_CGts mutation. In 3 tumor types (head&neck, liver, and myeloid cancer), NoCpG_CGtv appears to be the most prevalent sequence context (*p* < 0.05). In several tumor types such as brain and ovarian cancer, although NoCpG_CGtv did not act as the predominant mutation sequence context, it represented a fairly high percentage (brain: 32 % and ovarian: 35 %). However, in some tumor types such as bladder cancer, the hotspot mutations are significantly enriched in ATtv sequence context (35 %, *p* = 1.77e-2).

In terms of the specific sequence context that hotspot mutations occur across different tumor types, although insertion is not the most prevalent sequence context within breast cancer, the percentage of insertion in breast cancer (22 %) was significantly higher than in any other tumor types (*p* = 1.14e-02), similarly, the percentage of deletion in colorectal cancer (27 %) was obviously higher than in other tumor types (*p* = 1.84e-4), so as the percentage of ATts (36 %, *p* = 5.84e-3) in colorectal and ATtv (35 %, *p* = 3.73e-3) in myeloid cancer.

These observations revealed the common genomic features such as NoCpG_CGts and NoCpG_CGtv sequence context were positively selected across various tumor types as well as distinct genomic features that occurred in individual tumor types, and highlighted the significance of investigating the hotspot mutations under different sequence contexts separately to better understand their genetic complexities and functional indications.

To gain novel functional insight of these mutations that were predicted based on statistics of mutation data, we performed a set of additional statistical tests to associate these 702 hotspot mutations with functional evidences.

### Exploring the biological utilities of hotspot mutations using TCGA mRNA/protein expression data

The functional consequences of mutations may manifest in two aspects: affecting the gene expression or leading to abnormal signaling pathway activity. To address these questions, we divided the mRNA and protein expression values of a set of TCGA samples into multiple groups based on the mutational status of a specific gene in these samples: having a hotspot mutation, no hotspot mutation, or no mutations [22]. Only mutations occurring at least twice were included and Mann–Whitney U tests were used to measure the difference between different groups [23]. Among 702 hotspot mutations, we found 42 hotspot mutations resulted in significant mRNA or protein expression alterations (Additional file 8: Table S5).

**Fig. 4:**
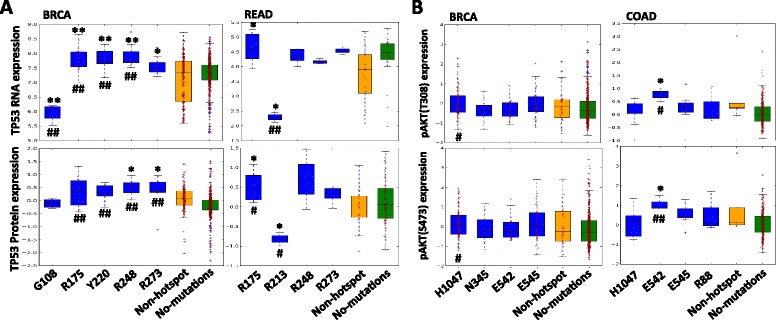
Functional implications of hotspot mutations in RNA and protein expression. **a** In BRCA, tumor samples with G108 deletion hotspot mutations in *TP53* exhibit lower *TP53* RNA expression than those with non-hotspot mutations and without *TP53* mutations. In contrast, tumor samples with missense hotspot mutations (R175, Y220, R248 and R273) in *TP53* show higher *TP53* RNA and protein expression. In READ, tumor samples with R175 missense mutations show higher *TP53* RNA and protein expression than those with non-hotspot mutations and without *TP53* mutations, while R213 nonsense mutations has the opposite effect. **b** In BRCA, tumor samples with H1047 missense hotspot mutations in *PIK3CA* show higher *AKT* pT308 and pS473 levels than those with no mutations in *PIK3CA*, while in COAD, tumor samples with E542 missense hotspot mutations in *PIK3CA* show higher *AKT* pT308 and pS473 levels than those with no mutations in *PIK3CA.* * indicates *p* < 0.05 and ** indicates *p* < 0.001 between samples with specified hotspot mutations and samples with non-hotspot mutations in examined gene; # indicates *p* < 0.05 and ## indicates *p* < 0.001 between samples with specified hotspot mutations and samples without mutations in examined gene

It is known that TP53 contains gain of function mutations associate with increased expression of TP53 [29, 30] through down-regulation of downstream targets such as *MDM2/MDM4,* which suppress the expression of *TP53*. However, it is not well investigated whether different mutations in *TP53* exhibit different functions across different cancer types. Motivated by this, we examined the association of *TP53* hotspot mutations and RNA and protein expression of *TP53* in different cancer types. To focus on the effect of mutations on *TP53* expression, we excluded samples harboring *TP53* deletions (Methods). As shown in Fig. 4a, in breast invasive carcinoma (BRCA), samples with R175, R248 and R273 missense mutations have obviously higher mRNA or protein expression levels, comparing to samples with non-hotspot mutations and with no mutation in *TP53*. In ovarian serous cystadenocarcinoma (OV), similar effects were observed for R248 and R273, which are associated with increases in the *TP53* mRNA and protein expressions (Additional file 10: Figure S5). However, in rectum adenocarcinoma (READ), although R175 is associated with increases in *TP53* RNA expressions similar to what is observed in BRCA, R248 and R273 missense mutations are not significantly associated with the *TP53* mRNA or protein expression, comparing to samples with non-hotspot or no mutations in *TP53* (Fig. 4a), implicating distinct functions of R248 and R273 in different disease contexts. In addition, G108 frame-shift deletion, I195 missense and R213 nonsense mutations, which were uniquely detected as hotspot mutations in BRCA, OV and READ respectively, are associated with either reduced or enhanced *TP53* expression in corresponding cancer types, suggesting the functional heterogeneity of hotspot mutations in different cancer types (Fig. 4a and Additional file 10: Figure S5).

Instead of altering the RNA/protein level, certain mutations may be functional via altering downstream protein activity through signaling transduction. For example, activation of *PIK3CA* could lead to activation of downstream targets such as *AKT* phosphorylation [31]. A set of *PIK3CA* mutations have been detected and functionally investigated in various cancer types such as BRCA and colon adenocarcinoma (COAD) [32]. We examined the association of individual *PIK3CA* mutations and *AKT* activation by comparing the phosphorylated *AKT* levels in samples with various *PIK3CA* mutations to those in samples without *PIK3CA* mutation. Surprisingly, in BRCA, only *PIK3CA* H1047 was associated with dramatically higher *AKT* pT308 and pS473 levels, comparing to those that did not have any *PIK3CA* mutations (Fig. 4b); in COAD, only *PIK3CA* E542 were associated with significantly higher *AKT* pT308 and pS473 levels, comparing to those that did not have any *PIK3CA* mutations (Fig. 4b). Notably, in both cases, *PIK3CA* mutations did not affect the total *AKT* level (data not shown), suggesting that different *PIK3CA* mutations in different cancer types may selectively activate *AKT* via signaling transduction, rather than expression regulation.

The availability of mRNA and protein expression data enable an opportunity to detailed characterize the biological consequences of different mutations in one cancer type, as well as one mutation under different cancer contexts, reiterating the rationale of distinguishing the function of individual mutations in different disease contexts.

### Exploring the pharmacogenomics properties of hotspot mutations

It has been shown that cancer cells respond to specific drugs when they harbor mutations in driver genes such as *BRAF* and *NRAS* [9]. However, it is not entirely clear whether different mutations in a driver gene can trigger different drug responses. Here, we assessed the effects of individual mutations on drug responsiveness using data from the CCLE [24]. We divided cancer cell-line samples into different groups, depending on whether they contain specific hotspot, non-hotspot, or no mutations in investigated gene candidates. Only mutations occurring at least twice were included and Mann–Whitney *U* test was performed to measure the difference [23]. Among 702 hotspot mutations, we found 35 hotspot mutations lead to significantly altered drug sensitivities (Additional file 8: Table S5).

**Fig. 5:**
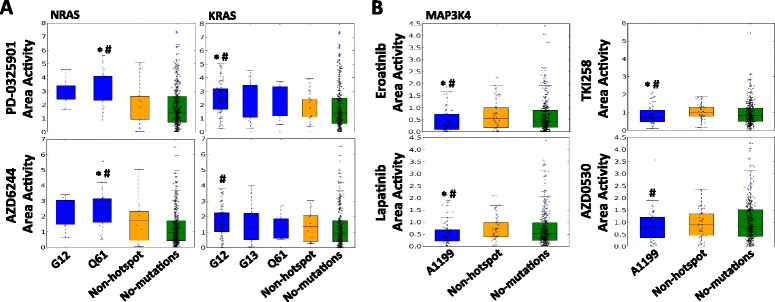
Functional implications of hotspot mutations in drug sensitivity. **a** Cancer cells with *NRAS* Q61 or *KRAS* G12 missense hotspot mutations exhibit higher sensitivity to MEK inhibitors (PD-0325901 and AZD6244) than those with non-hotspot mutations or without any mutations in *NRAS or KRAS*. **b** Cancer cells with *MAP3K4* A1199 deletion hotspot mutations exhibit lower sensitivity to different EGFR inhibitors (Erlotinib, Lapatinib, TKI258 and AZD0530) than those with non-hotspot mutations or without any mutations in *MAP3K4*. * indicates *p* < 0.05 between samples with specified hotspot mutations and samples with non-hotspot mutations in examined gene; # indicates *p* < 0.05 between samples with specified hotspot mutations and samples without mutations in examined gene

We first illustrated the effect of individual hotspot mutations in *BRAF, KRAS* and *NRAS* on the sensitivity of cancer cells treated by *MEK* inhibitors (PD-0325901 and AZD6244). As expected, cells with *BRAF* V600E mutations demonstrated significantly higher sensitivity to *MEK* inhibitors than those without *BRAF* mutations (data not shown). Furthermore, we found that cells with *NRAS* Q61 hotspot mutations demonstrated significantly higher sensitivity to *MEK* inhibitors than those with non-hotspot mutations and those without mutations in *NRAS* (Fig. 5a). Cells with *KRAS* G12 hotspot mutations demonstrated significantly higher sensitivity to *MEK* inhibitors than those with non-hotspot mutations and those without mutations in *KRAS* (Fig. 5a).

Epidermal growth factor (*EGF*) is one of the high affinity ligands of *EGFR. EGF/EGFR* system induces cell growth, differentiation, migration, adhesion and cell survival through various interacting signaling pathways such as *MAPK* pathway [33], in which *MAP3K4* is an important component [34]. Clinically, *EGFR* inhibitors such as Erlotinib were used to repress *EGFR* signaling activations and suppress tumor cell growth. However, we found that cancer cell-lines with *MAP3K4* A1199 deletion hotspot mutations were more resistant to all four examined *EGFR* inhibitors (Erlotinib, Lapatinib, TKI258 and AZD0530) in comparison to cancer cell-lines without *MAP3K4* mutations (Fig. 5b). These *EGFR* hotspot mutant cell-lines are also more resistant to three inhibitors (Erlotinib, Lapatinib and TKI258) in comparison to cell-lines containing non-hotspot mutations in *MAP3K4* (Fig. 5b), suggesting the unique function of *MAP3K4* A1199 deletion in disrupting the *MAPK* pathway function and its potential biomarker utility.

These observations above support that hotspot mutations we identified may have distinct roles in mediating signaling pathways and are associated with different drug sensitivities. Therefore, it is critical to obtain accurate genomic information and interpret them in context-specific manner in order to achieve desirable outcomes in personalized cancer treatment.

### Tumor type-specific hotspot mutations

We performed an analysis to assess whether a hotspot mutation in our set is highly prevalent in specific tumor types. Among all the 702 hotspots, we found that 68 were highly prevalent in one tumor type, 11 in two tumor types, 2 (*KRAS* G12 and *PIK3CA* E542) in three tumor types, and 1 (*KRAS* G13) in four tumor types (Additional file 11: Figure S6). Among these, 34 hotspot mutations such as *CD209* R129 missense (4.0 %) in bladder cancer, *MAGI1* Q421 insertion (0.8 %) and *NR1H2* Q175 insertion (1.8 %) in breast cancer were not well investigated based on previous studies and are potentially novel targets (Additional file 8: Table S5).

**Fig. 6:**
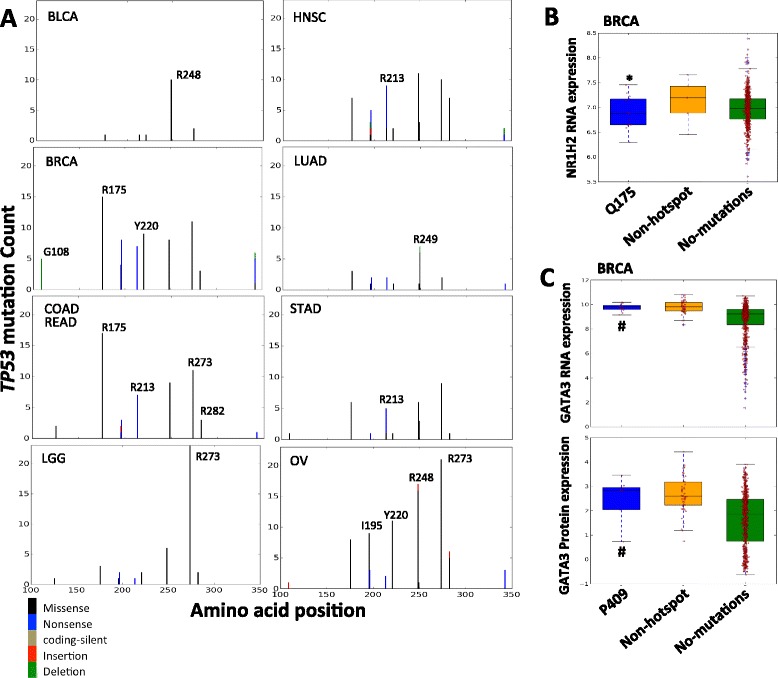
Prevalence of hotspot mutations in different TCGA cancer types and their functional implications. **a** In *TP53*, hotspot mutations are differentially prevalent in different tumor types, indicating their differential functions. **b** In BRCA, samples with *NR1H2* Q175 in-frame insertion hotspot mutations have significantly lower NR1H2 expression compared to samples with *NR1H2* non-hotspot mutations. **c** In BRCA, sample with *GATA3* P409 insertion hotspot mutations have obviously higher GATA3 compared to samples without GATA3 mutation. * indicates *p* < 0.05 between samples with specified hotspot mutations and samples with non-hotspot mutations in examined gene; # indicates *p* < 0.05 between samples with specified hotspot mutations and samples without mutations in examined gene

Of the 21 hotspot mutations detected in *TP53* (Fig. 6a), 2 were found to be prevalent in multiple cancer types (R248 in bladder urothelial carcinoma (BLCA), BRCA and OV, R273 in lower grade glioma (LGG), BRCA and OV), and 9 (G108, R158, R175, I195, R213, Y220, R249, R282, E285) in one tumor type, confirming the functional diversity of *TP53* hotspot mutations in different cancer types (Fig. 4a).

We identified 30 hotspot mutations that were exclusively detected in only one tumor type (Additional file 12: Table S6). Included were *DNMT3A* R882 and *NPM1* W288, which occur in 14.9 and 25.6 % of acute myeloid leukemia (LAML) patients, respectively and have been shown important in LAML oncogenesis [35]. Besides these expected hotspots, we found some potentially novel hotspots. For example, we found an in-frame insertion hotspot mutation, *NR1H2* Q175 in 1.8 % of BRCA patients, further investigation using BRCA mRNA expression data showed that *NR1H2* Q175 insertion is associated with reduced mRNA expression of *NR1H2*, comparing to *NR1H2* non-hotspot mutations (Mann–Whitney *U* test, *p* = 2.60e-2, Fig. 6b). Although having been reported to regulate cholesterol homeostasis and tumorigenesis of liver cancer [36], the role of *NR1H2* Q175 insertion in BRCA has not been well characterized. In addition, *GATA3* P409, a frame-shift insertion hotspot mutation was detected in 1.6 % of BRCA patients. BRCA samples with *GATA3* P409 insertions had higher expressions of *GATA3* compared to samples without *GATA3* mutations based on both the BRCA mRNA expression (Mann–Whitney *U* test, *p* = 2.03e-2) and RRPA data (Mann–Whitney *U* test, *p* = 5.94e-2, Fig. 6c). Because *GATA3* has been proposed as a prognostic biomarker in breast cancer [37], the high frequency of *GATA3* P409 and elevated *GATA3* expression in BRCA make it a potential useful therapeutic target in clinics.

### Conservation and protein-domain characteristics of the hotspot mutations

**Fig. 7:**
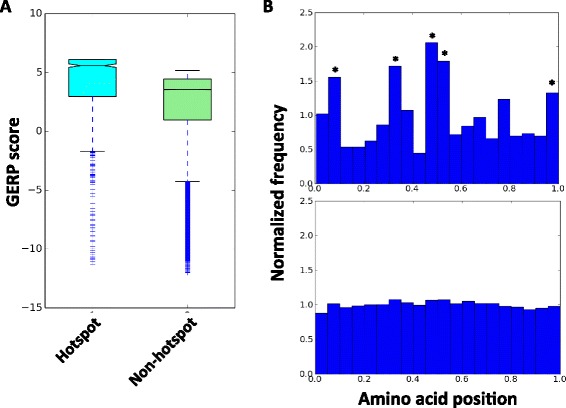
Compare the conservation and proteomic domain localization of the hotspot and the non-hotspot mutations. **a** Comparison of GERP score between the hotspot and non-hotspot mutations. **b** Investigation of the proteomic domain location of the hotspot (*upper*) and non-hotspot (*lower*) mutations

In general, functional and structural important mutations are expected to locate in highly evolutionally conserved region and domain in the protein. To evaluate our hotspot mutation, we used the RS scores computed by GERP++ [25], to measure the evolutionary constraints across different chromosomal sites (Methods). We compared the RS score difference between the sites that belong to hotspot mutations and those belong to non-hotspot mutations. The RS scores of 702 hotspot mutations were significantly higher than those of non-hotspot mutations (Fig. 7a), suggesting the sites that harbor hotspot mutations were more conserved than those do not. In addition, we also examined the relative location of mutations on the protein. The non-hotspot mutations were evenly distributed across different domains of the protein (lower panel), while the hotspot mutations showed clustering in the middle and the terminals (Fig. 7b, upper panel), suggesting the functional preference of mutations in different protein domains.

## Discussion

We nominated 702 hotspot mutations in 549 genes from the COSMIC database, among which 53 were associated with statistically significant functional evidences in currently available TCGA and CCLE data (Additional file 8: Table S5). The rest of the hotspot mutations could not be associated with additional functional evidence, which may due to sparseness in the data and limitations in the current knowledge bases. For example, only 187 proteins were available on the RPPA, the sample size was relatively small and some observed patterns might change as the sample size increases in the future. Nonetheless, our study revealed differential biological consequences and pharmacogenomics utilities of mutations under different disease contexts and highlighted the significance of allocating the specific function of individual mutations using functional genomics and pharmacogenomics data. These aspects have not been systematically explored in previous studies. Besides investigating previous known hotspot mutations in different contexts, we also nominated a set of novel hotpot mutations such as those in *MAP3K4, NR1H2* and *GATA3* with corresponding functional associations, represents good candidates for developing predictive biomarkers and drug targets.

Investigate the mutational signature in different cancer types has been a significant action to understand the underlying biological processes of cancer development. Alexandrov et al. [14] dissected all the mutations into 21 distinct mutational signatures with diverse sequence contexts enrichment and associated them with different phenotypes such as age of the patient at cancer diagnosis, known mutagenic exposures or defects in DNA maintenance. Kandoth et al. [13] investigated the 12 cancer types in TCGA and reported that mutations were enriched in C/G transitions such as C- > T and C/G transversions such as C- > A in different cancer types using all the mutation data. In our study, we focused on predicted hotspot mutations and illustrated the mutational signatures that hotspot mutations represented. We found that hotspot mutations were enriched in NoCpG_CGts and NoCpG_CGtv sequence context in 10 tumor types and some sequence contexts such as ATtv in bladder cancer. In addition, we elucidated that insertion mutations were highly enriched in breast cancer and deletion mutations were enriched in colorectal cancer, which was a novel founding in our study.

Another novel contribution of our current investigation was to highlight the criticalness of distinguishing the biological roles of individual hotspot mutations within one cancer gene under different disease contexts. Different hotspot mutations within one gene can exhibit diverse functional indications. For example, only *PIK3CA* H1047 but not any other hotspot mutations enhances the *AKT* pathway activity in BRCA, while only *PIK3CA* E542 enhances the *AKT* pathway activity in COAD. Previous studies observed that *PIK3CA* H1047R and E545K both result in a constitutively active enzyme with oncogenic capacity but the effect of H1047R is much stronger than E545K [32, 38, 39]. In our analysis, We did observe enhanced AKT pathway activity in tumor samples containing E545K. However, the difference was not significant due likely to 1) insufficient sample size that carrying the *PIK3CA* E545K mutation or 2) highly sparse expression of phosphor-AKT in samples without *PIK3CA* mutation in TCGA samples. Similarly, one hotspot mutation can represent different functional relevance in different cancer types. For example, *TP53* R248 and R273 significantly increase its RNA and protein expression in BRCA and OV but not in READ. In addition, different *TP53* hotspot mutations were prevalence in various cancer types, and 30 hotspot mutations exclusively occur in only one cancer type.

Along the line of identifying hotspot mutations, it was commonly assumed that mutations close to each other are expected to exhibit similar functions and grouping nearby mutations as a hotspot would improve the power of identifying driver mutations. One important observation of our study was we found that even hotspot mutations close to each other could have distinct biological implications in the same cancer type. For example, *PIK3CA* E542 was significantly associated with enhancement of phospho-AKT activities in COAD, while E545 did not; cell-lines with *KRAS* G13 were resistant to IGF-1R inhibitor (AEW541), while those with G12 did not (data not shown). Nearby hotspot mutations demonstrated distinct functions under different disease context. Simply collapsing mutations based on proximity and assuming that nearby mutations have the same functions may result in errors in functional prediction.

Although available functional genomic data prohibited us from systematic uniformly characterizing every hotspot mutation we predicted, our integrative assessment based on mRNA expression, protein activity, drug sensitivity, and tumor specificity data in TCGA and CCLE, indicated potential utility of each of our predicted hotspot mutations. Such functional characterization can be unequivocally improved in the future by using systematic pathway-aware algorithms such as DriverNet [40] and PARADIGM-SHIFT [41], and by integrating additional functional genomic datasets such as Genomics of Drug Sensitivity in Cancer (GDSC) [42]. In terms of identifying hotspot mutations in the amino acid level, it is critical to have consistent annotations from genomic to protein level. Zhou, et al. [43] used COSMIC data to show that ambiguities frequently exist in variant annotation, and annotation tool such as TransVar [43] would be very helpful to improve the accuracy of hotspot mutation prediction. In addition, further dissecting the mutation data into different cancer subtype groups (such as MSI and non-MSI in colorectal cancer, ER+, HER2+ and TNBC in breast cancer) would be helpful to distinguish distinct mutation profiles and precisely investigate the specific function of hotspot mutations in different cancer subtypes. Importantly, our results demonstrated a high degree of functional heterogeneity at the mutational level, which has not been sufficiently apprehended or investigated in current research and clinical practice. Despite all the caveats, the hotspot mutations we identified provide a step forward in cataloging hotspot driver mutations in different cancer types and biological contexts, which is critical for realizing the promise of personalized cancer medicine.

## Conclusion

We observed a large discordancy of mutation rates across different mutation subtypes and tumor types, and nominated 702 hotspot mutations in 549 cancer genes using COSMIC data in a gene, tumor type, mutation subtype and sequence context specific manner. We illustrated the common and distinct mutational signatures of hotspot mutations across different tumor types and employed multi-dimensional functional evidences to demonstrate the diverse functional relevance of hotspot mutations in different biological and disease contexts and nominate novel hotspot mutations such as *MAP3K4* A1199 deletion, *NR1H2* R175 insertion, and *GATA3* P409 insertion with functional associations. Our results will promote our understanding of the process of genomic positive selection by investigating the mutational signatures on hotspot mutations and facilitate ongoing efforts in cancer target discovery and development [44]. The source code used for our analysis is available at https://sourceforge.net/projects/hotdriver/.

### Ethics approval and consent to participate

Not Applicable.

### Consent for publication

Not Applicable.

### Availability of data and material

The datasets supporting the conclusions of this article are included within the article and its additional files.

## Additional files

Additional file 1: Table S1. Number of samples in 17 tumor types in COSMIC v71. (PDF 57 kb) Additional file 2: Table S2. Twenty mutation subtypes that were included in the statistical modeling of hotspot mutation definition. (PDF 34 kb) Additional file 3: Table S3. Number of samples available in different TCGA cancer types. (PDF 66 kb) Additional file 4: Table S4. 2 × 2 table of calculating the prevalence of target mutation *B* in samples *A*. (PDF 46 kb) Additional file 5: Figure S1. The percentage of different mutational subtypes across all defined hotspot mutations. On each hotspot locus, only the mutational subtype that occupies the highest number of mutations was counted. (PDF 85 kb) Additional file 6: Figure S2. The significance of overlap (y-axis, calculated using Fisher exact test) between hotspot-mutation-containing-genes and previously known cancer genes at various adjusted *p* value cutoffs (x-axis). (PDF 37 kb) Additional file 7: Figure S3. Number of hotspot mutations identified in individual tumor types using COSMIC data. (PDF 183 kb) Additional file 8: Table S5 List of the predicted hotspot mutations in different tumor types based on COSMIC version 71. Functional evidences were annotated if the statistical analysis showed significant results. (XLSX 145 kb) Additional file 9: Figure S4. Relationship between the number of hotspot mutations and the total number of mutations (mutation burden) in each tumor type. (PDF 79 kb) Additional file 10: Figure S5. Functional implications of hotspot mutations in RNA and protein expression. In OV, tumor samples with missense hotspot mutations (I195, Y220, R248 and R273) in *TP53* show higher *TP53* RNA and protein expression than those with non-hotspot mutations and without *TP53* mutations. * indicates *p* < 0.05 and ** indicates *p* < 0.001 between samples with specified hotspot mutations and samples with non-hotspot mutations in examined gene; # indicates *p* < 0.05 and ## indicates *p* < 0.001 between samples with specified hotspot mutations and samples without mutations in examined gene. (PDF 365 kb) Additional file 11: Figure S6. Prevalence of hotspot mutations in different TCGA cancer types. 82 hotspot mutations were highly prevalent in one or more cancer types. Most are highly prevalent in only one tumor type, while a few were in two or more tumor types. (PDF 44 kb) Additional file 12: Table S6. Hotspot mutations exclusively detected in only one tumor type in TCGA pan-cancer data. (PDF 70 kb)
