# Supplementary material for: Hotspot mutations delineating diverse mutational signatures and biological utilities across cancer types
Source: BMC Genomics. 2016 Jun 23;17(Suppl 2):394. doi: 10.1186/s12864-016-2727-x (PMC4928158; doi:10.1186/s12864-016-2727-x)
Supplement: Additional file 1: Table S1. — Number of samples in 17 tumor types in COSMIC v71. (PDF 57 kb) [file 12864_2016_2727_MOESM1_ESM.pdf]

Additional file 1: Table S1 Number of samples in 17 tumor types in COSMIC v71

Tumor Type	COSMIC samples *	WGS&WEX ^	Exclude Hyper-mutator #
**Bladder**	3872	364	358
**Bone**	704	81	79
**Brain**	8457	1366	1354
**Breast**	4994	1152	1140
**Colon**	29413	694	684
**Endometrium**	2293	271	260
**Head&Neck**	3036	710	699
**Kidney**	3616	879	867
**Liver**	2448	900	890
**Lung**	10520	969	951
**Myeloid**	52500	1344	1336
**Ovarian**	3378	647	640
**Pancreas**	5561	800	789
**Prostate**	953	508	501
**Skin**	9072	655	650
**Stomach**	3615	621	613
**Thyroid**	13967	444	439

* Number of samples that were collected by COSMIC v71; ^ Number of samples that were subjected to either whole genome or whole exome sequencing; # Number of samples after excluding samples that were shown to be hyper-mutated.
