# Supplementary material for: Hotspot mutations delineating diverse mutational signatures and biological utilities across cancer types
Source: BMC Genomics. 2016 Jun 23;17(Suppl 2):394. doi: 10.1186/s12864-016-2727-x (PMC4928158; doi:10.1186/s12864-016-2727-x)
Supplement: Additional file 2: Table S2. — Twenty mutation subtypes that were included in the statistical modeling of hotspot mutation definition. (PDF 34 kb) [file 12864_2016_2727_MOESM2_ESM.pdf]

Additional file 2: Table S2 20 mutation subtypes that were included in the statistical modeling of hotspot mutation definition

Mutation_subtype
Missense A/T transition
Missense A/T transversion
Missense non-CpG C/G transition
Missense non-CpG C/G transversion
Missense CpG C/G transition
Missense CpG C/G transversion
Nonsense A/T transition
Nonsense A/T transversion
Nonsense non-CpG C/G transition
Nonsense non-CpG C/G transversion
Nonsense CpG C/G transition
Nonsense CpG C/G transversion
Silent A/T transition
Silent A/T transversion
Silent non-CpG C/G transition
Silent non-CpG C/G transversion
Silent CpG C/G transition
Silent CpG C/G transversion
Insertion
Deletion
