# Supplementary material for: Hotspot mutations delineating diverse mutational signatures and biological utilities across cancer types
Source: BMC Genomics. 2016 Jun 23;17(Suppl 2):394. doi: 10.1186/s12864-016-2727-x (PMC4928158; doi:10.1186/s12864-016-2727-x)
Supplement: Additional file 3: Table S3. — Number of samples available in different TCGA cancer types. (PDF 66 kb) [file 12864_2016_2727_MOESM3_ESM.pdf]

Additional file 3: Table S3 Number of samples available in different TCGA cancer types.

Abbreviation	Tumor_Type	Mutation	RNA	RPPA	Mutation&RNA	Mutation&RPPA
**BRCA**	Breast Invasive Carcinoma	772	817	748	752	637
**KIRC**	Kidney Renal Clear Cell Carcinoma	417	470	455	391	386
**THCA**	Thyroid Carcinoma	323	426	NA	303	NA
**OV**	Ovarian Serous Cystadenocarcinoma	316	263	413	163	210
**HNSC**	Head & Neck Squamous Cell Carcinoma	306	303	213	299	208
**GBM**	Glioblastoma Multiforme	291	161	216	150	146
**SKCM**	Skin Cutaneous Melanoma	253	NA	NA	NA	NA
**UCEC**	Uterine Corpus Endometrioid Carcinoma	248	333	404	239	203
**LUAD**	Lung Adenocarcinoma	230	353	238	169	135
**COAD-READ**	Colon & Rectum Adenocarcinoma	224	263	466	217	157
**LAML**	Acute Myeloid Leukemia	194	173	NA	169	NA
**LUSC**	Lung Squamous Cell Carcinoma	178	220	196	177	112
**LGG**	Brain Lower Grade Glioma	170	205	NA	166	NA
**STAD**	Stomach Adenocarcinoma	151	58	NA	58	NA
**KIRP**	Kidney Renal Papillary Cell Carcinoma	100	78	NA	77	NA
**BLCA**	Bladder Urothelial Carcinoma	99	96	128	95	92
**PRAD**	Prostate Adenocarcinoma	83	142	NA	72	NA
**CESC**	Cervical Squamous Cell Carcinoma	39	97	NA	38	NA
**PAAD**	Pancreatic Adenocarcinoma	34	41	NA	19	NA

Note: The number of samples with somatic mutation data, RNA expression data, RPPA data, or both data types (Mutation&RNA and Mutation&RPPA) in each TCGA cancer type.
