# Supplementary material for: Hotspot mutations delineating diverse mutational signatures and biological utilities across cancer types
Source: BMC Genomics. 2016 Jun 23;17(Suppl 2):394. doi: 10.1186/s12864-016-2727-x (PMC4928158; doi:10.1186/s12864-016-2727-x)
Supplement: Additional file 4: Table S4. — 2 × 2 table of calculating the prevalence of target mutation B in samples A. (PDF 46 kb) [file 12864_2016_2727_MOESM4_ESM.pdf]

Additional file 4: Table S4 2×2 table of calculating the prevalence of target mutation ***B*** in samples ***A***

Number of ***A*** samples with ***B*** mutations	Number of ***A*** samples without ***B*** mutations
Number of non-***A*** samples with ***B*** mutations	Number of non-***A*** samples without ***B*** mutations
