# Supplementary material for: Hotspot mutations delineating diverse mutational signatures and biological utilities across cancer types
Source: BMC Genomics. 2016 Jun 23;17(Suppl 2):394. doi: 10.1186/s12864-016-2727-x (PMC4928158; doi:10.1186/s12864-016-2727-x)
Supplement: Additional file 5: Figure S1. — The percentage of different mutational subtypes across all defined hotspot mutations. On each hotspot locus, only the mutational subtype that occupies the highest number of mutations was counted. (PDF 85 kb) [file 12864_2016_2727_MOESM5_ESM.pdf]

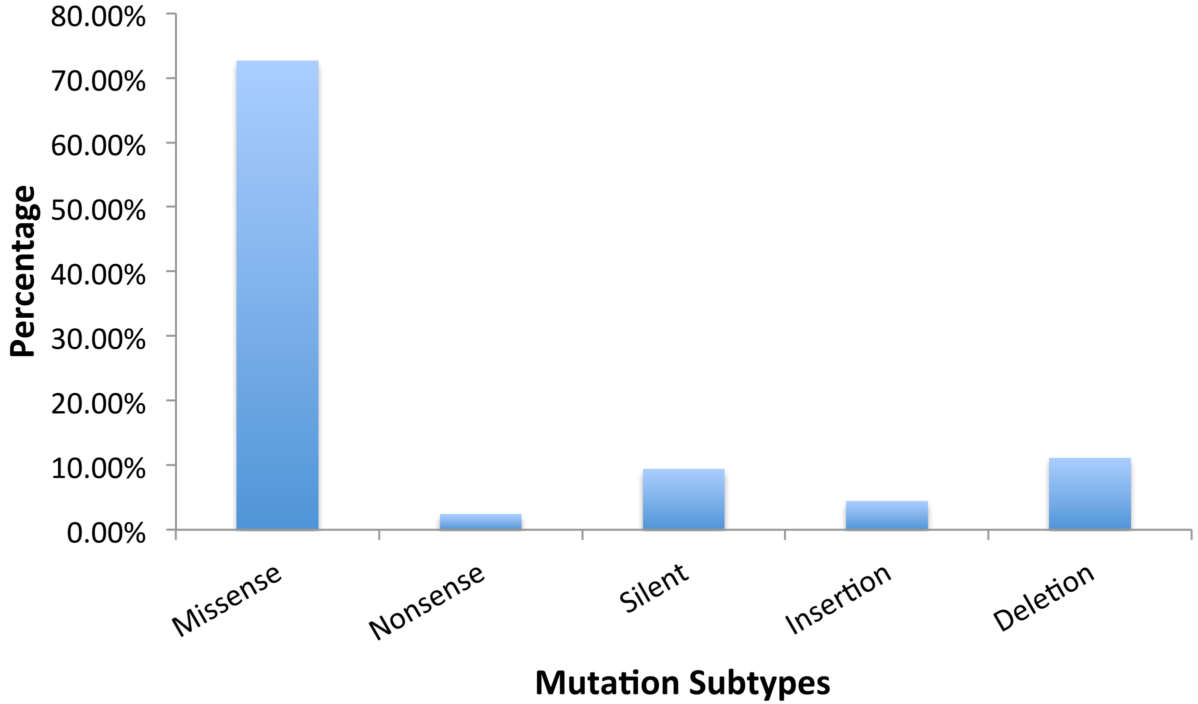

Additional file 5: Figure S1 The percentage of different mutational subtypes across all defined hotspot mutations. On each hotspot locus, only the mutational subtype that occupies the highest number of mutations was counted.
