# Supplementary material for: Hotspot mutations delineating diverse mutational signatures and biological utilities across cancer types
Source: BMC Genomics. 2016 Jun 23;17(Suppl 2):394. doi: 10.1186/s12864-016-2727-x (PMC4928158; doi:10.1186/s12864-016-2727-x)
Supplement: Additional file 6: Figure S2. — The significance of overlap (y-axis, calculated using Fisher exact test) between hotspot-mutation-containing-genes and previously known cancer genes at various adjusted p value cutoffs (x-axis). (PDF 37 kb) [file 12864_2016_2727_MOESM6_ESM.pdf]

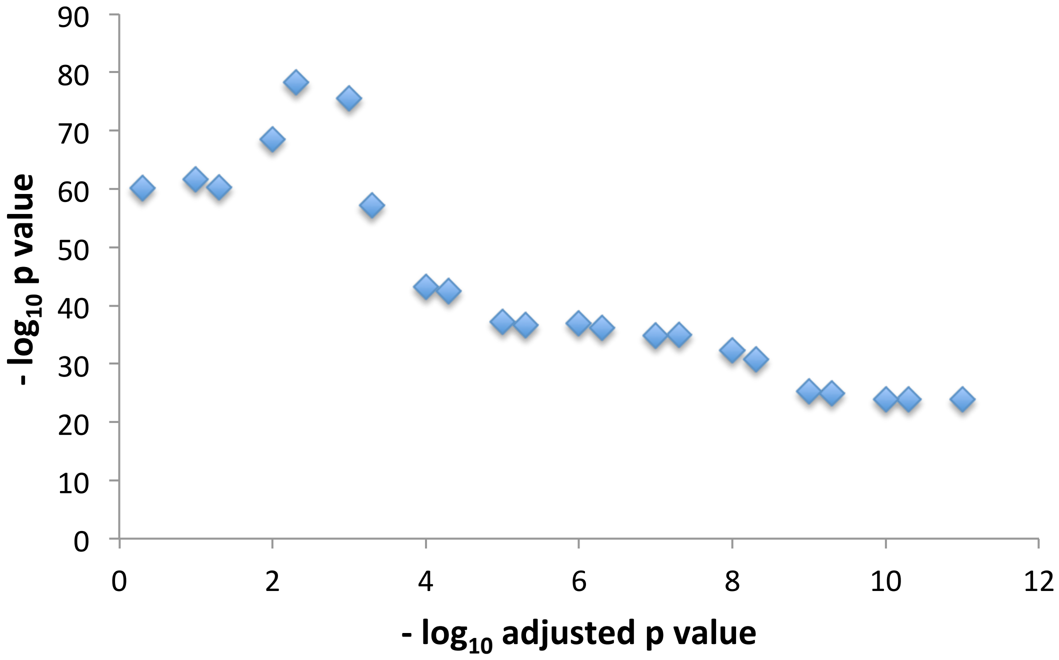

Additional file 6: Figure S2 The significance of overlap (y-axis, calculated using Fisher exact test) between hotspot-mutation-containing-genes and previously known cancer genes at various adjusted p value cutoffs (x-axis).
