# Supplementary material for: Hotspot mutations delineating diverse mutational signatures and biological utilities across cancer types
Source: BMC Genomics. 2016 Jun 23;17(Suppl 2):394. doi: 10.1186/s12864-016-2727-x (PMC4928158; doi:10.1186/s12864-016-2727-x)
Supplement: Additional file 7: Figure S3. — Number of hotspot mutations identified in individual tumor types using COSMIC data. (PDF 183 kb) [file 12864_2016_2727_MOESM7_ESM.pdf]

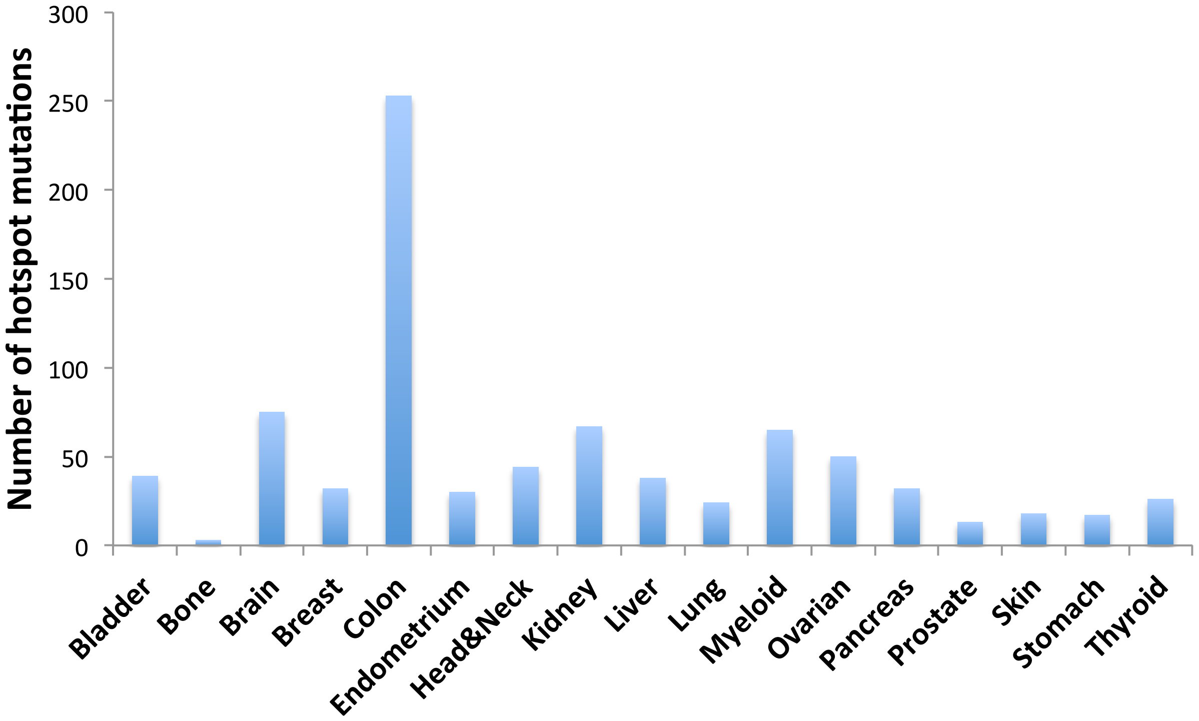

Additional file 7: Figure S3 Number of hotspot mutations defined in individual tumor types using COSMIC data.
