# Supplementary material for: Hotspot mutations delineating diverse mutational signatures and biological utilities across cancer types
Source: BMC Genomics. 2016 Jun 23;17(Suppl 2):394. doi: 10.1186/s12864-016-2727-x (PMC4928158; doi:10.1186/s12864-016-2727-x)
Supplement: Additional file 9: Figure S4. — Relationship between the number of hotspot mutations and the total number of mutations (mutation burden) in each tumor type. (PDF 79 kb) [file 12864_2016_2727_MOESM9_ESM.pdf]

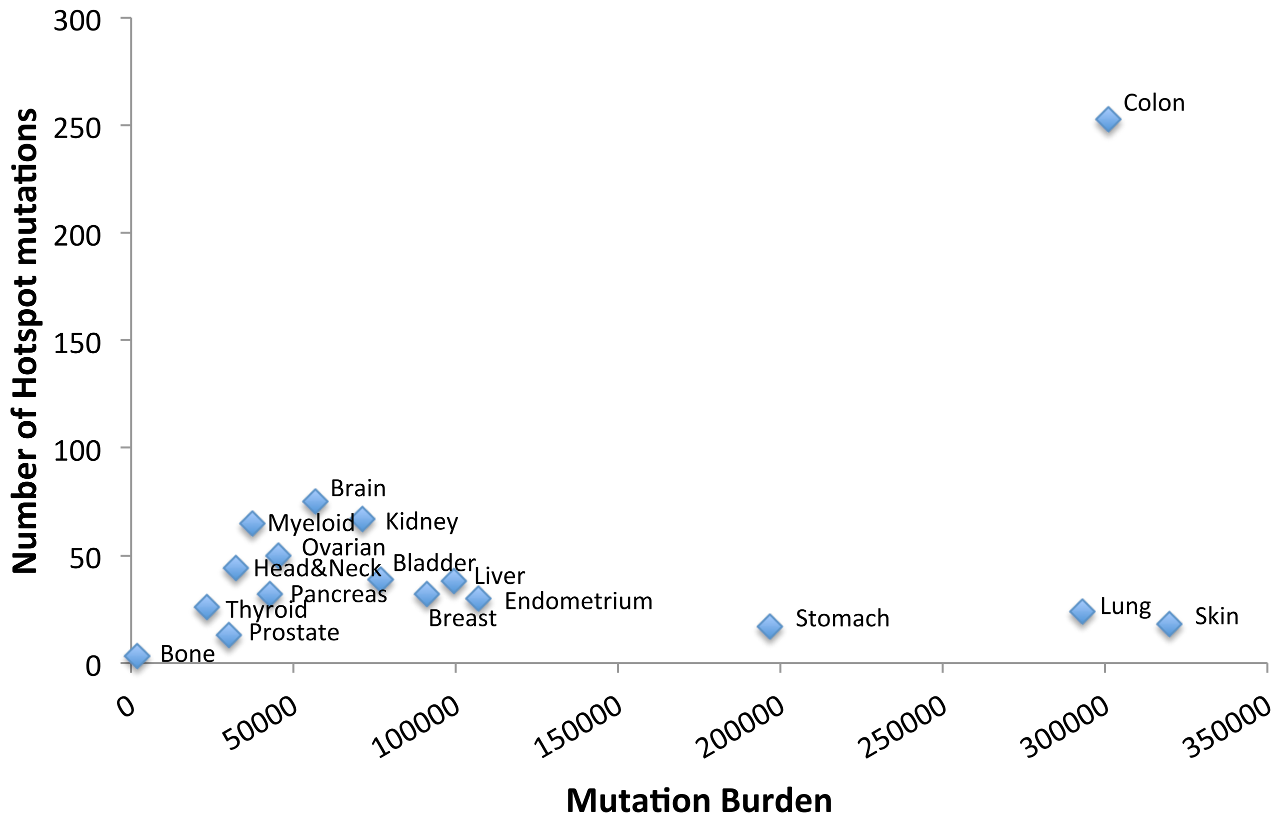

Additional file 9: Figure S4 Relationship between the number of hotspot mutations and the total number of mutations (mutation burden) in each tumor type.
