# Supplementary material for: Hotspot mutations delineating diverse mutational signatures and biological utilities across cancer types
Source: BMC Genomics. 2016 Jun 23;17(Suppl 2):394. doi: 10.1186/s12864-016-2727-x (PMC4928158; doi:10.1186/s12864-016-2727-x)
Supplement: Additional file 10: Figure S5. — Functional implications of hotspot mutations in RNA and protein expression. In OV, tumor samples with missense hotspot mutations (I195, Y220, R248 and R273) in TP53 show higher TP53 RNA and protein expression than those with non-hotspot mutations and without TP53 mutations. * indicates p < 0.05 and ** indicates p < 0.001 between samples with specified hotspot mutations and samples with non-hotspot mutations in examined gene; # indicates p < 0.05 and ## indicates p < 0.001 between samples with specified hotspot mutations and samples without mutations in examined gene. (PDF 365 kb) [file 12864_2016_2727_MOESM10_ESM.pdf]

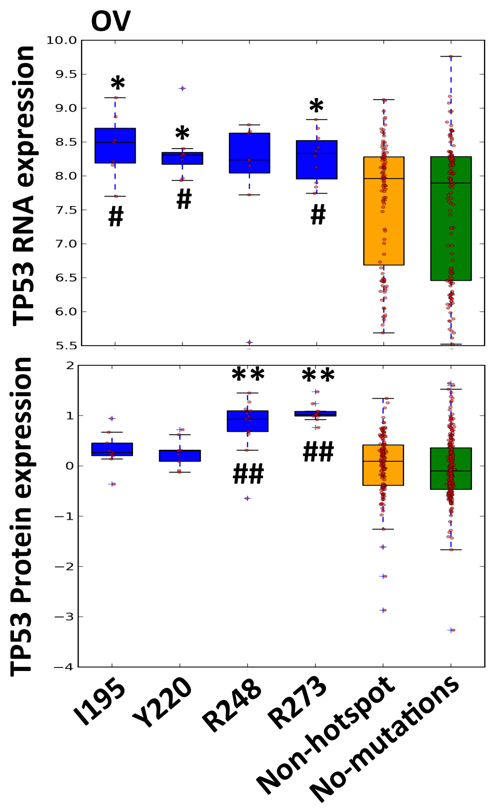

Additional file 10: Figure S5 Functional implications of hotspot mutations in RNA and protein expression. In OV, tumor samples with missense hotspot mutations (I195, Y220, R248 and R273) in *TP53* show higher *TP53* RNA and protein expression than those with non-hotspot mutations and without *TP53* mutations. * indicates p<0.05 and ** indicates p<0.001 between samples with specified hotspot mutations and samples with non-hotspot mutations in examined gene; # indicates p<0.05 and ## indicates p<0.001 between samples with specified hotspot mutations and samples without mutations in examined gene.
