# Supplementary material for: Hotspot mutations delineating diverse mutational signatures and biological utilities across cancer types
Source: BMC Genomics. 2016 Jun 23;17(Suppl 2):394. doi: 10.1186/s12864-016-2727-x (PMC4928158; doi:10.1186/s12864-016-2727-x)
Supplement: Additional file 11: Figure S6. — Prevalence of hotspot mutations in different TCGA cancer types. 82 hotspot mutations were highly prevalent in one or more cancer types. Most are highly prevalent in only one tumor type, while a few were in two or more tumor types. (PDF 44 kb) [file 12864_2016_2727_MOESM11_ESM.pdf]

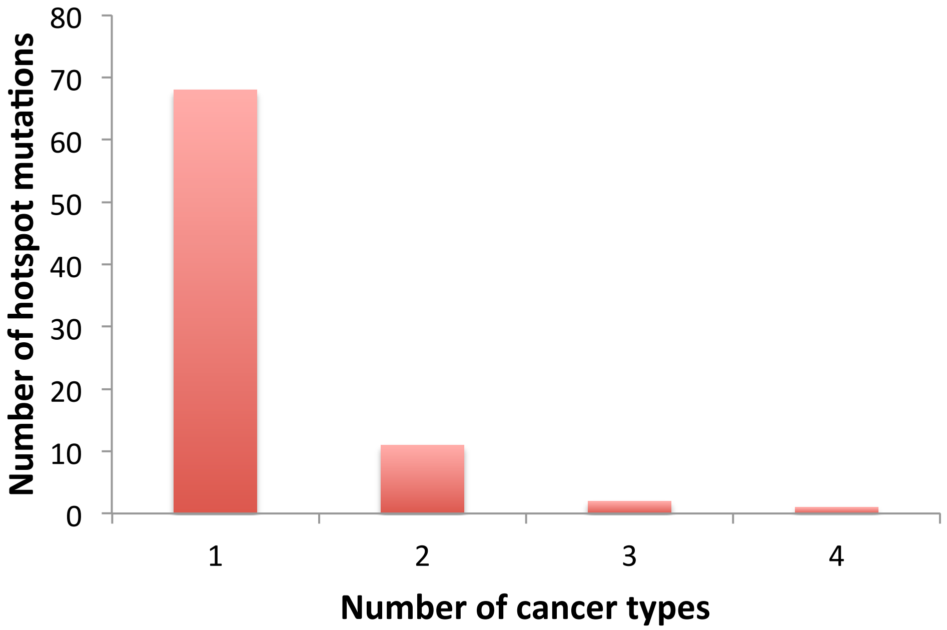

Additional file 11: Figure S6 Prevalence of hotspot mutations in different TCGA cancer types. 82 hotspot mutations were highly prevalent in one or more cancer types. Most are highly prevalent in only one tumor type, while a few were in two or more tumor types.
