# Supplementary material for: Hotspot mutations delineating diverse mutational signatures and biological utilities across cancer types
Source: BMC Genomics. 2016 Jun 23;17(Suppl 2):394. doi: 10.1186/s12864-016-2727-x (PMC4928158; doi:10.1186/s12864-016-2727-x)
Supplement: Additional file 12: Table S6. — Hotspot mutations exclusively detected in only one tumor type in TCGA pan-cancer data. (PDF 70 kb) [file 12864_2016_2727_MOESM12_ESM.pdf]

Additional file 12: Table S6 Hotspot mutations exclusively detected in only one tumor type in TCGA pan-cancer data.

Tumor_Type	Gene	aaPosition	Frequency	Adj.Pvalue
BLCA	RXRA	S427	5.00%	4.26E-08
BRCA	GATA3	P409	1.60%	5.48E-09
BRCA	GOLGA6L2	E537	0.90%	1.27E-04
BRCA	MAGI1	Q421	0.80%	4.95E-04
COAD&READ	APC	R876	5.30%	4.01E-14
KIRC	NEFH	P655	0.70%	2.16E-03
LAML	FLT3	D835	8.20%	1.94E-21
LAML	NPM1	W288	25.60%	1.58E-69
LAML	DNMT3A	R882	14.90%	4.24E-38
SKCM	AGAP10	M293	2.80%	1.62E-08
SKCM	C15orf23	S24	2.80%	1.62E-08
SKCM	PCDHGA1	R293	2.00%	3.48E-06
SKCM	TRRAP	S722	1.60%	4.83E-05
STAD	BMPR2	N583	2.00%	1.49E-04
STAD	CCDC43	R216	2.00%	1.49E-04
STAD	ESRP1	N512	3.30%	3.25E-07
STAD	FAM18A	F140	2.00%	1.49E-04
STAD	GTF2I	N440	2.00%	1.49E-04
STAD	STAMBPL1	K405	2.00%	1.49E-04
STAD	ZNF365	K399	2.00%	1.49E-04
STAD	CNBD1	L396	2.00%	4.68E-04
STAD	DOCK3	P1852	6.60%	2.47E-13
STAD	PGM5	I98	10.50%	5.36E-22
STAD	SLC3A2	K331	2.60%	3.15E-05
STAD	UBR5	E2121	5.30%	1.72E-10
UCEC	FGFR2	S252	3.60%	6.31E-11
UCEC	MAX	H28	1.60%	4.55E-05
UCEC	BCOR	N1459	3.20%	7.14E-09
UCEC	PIK3CA	R93	2.40%	1.25E-06

Note: Frequency was calculated by dividing number of mutations over number of samples in specific tumor type; adjusted p-value was computed based on Fisher's exact test followed by FDR correction.
